# Iatrogenic Blood-borne Viral Infections in Refugee Children from War and Transition Zones

**DOI:** 10.3201/eid1906.120806

**Published:** 2013-06

**Authors:** Paul N. Goldwater

**Affiliations:** Women’s and Children’s Hospital, Adelaide, South Australia, Australia;; University of Adelaide, Adelaide

**Keywords:** HIV, hepatitis B virus, hepatitis C virus, iatrogenic, children, war, pathogenesis, viruses, infections, transfusions

## Abstract

Pediatric infectious disease clinicians in industrialized countries may encounter iatrogenically transmitted HIV, hepatitis B virus, and hepatitis C virus infections in refugee children from Central Asia, Southeast Asia, and sub-Saharan Africa. The consequences of political collapse and/or civil war—work migration, prostitution, intravenous drug use, defective public health resources, and poor access to good medical care—all contribute to the spread of blood-borne viruses. Inadequate infection control practices by medical establishments can lead to iatrogenic infection of children. Summaries of 4 cases in refugee children in Australia are a salient reminder of this problem.

Blood-borne viruses (BBVs) have benefitted from internal political strife, migration, prostitution, intravenous/injection drug use, and defective public health resources in some Central Asian republics and Southeast Asian and sub-Saharan African countries. Iatrogenic transmission of HIV in children in Romania (*1*) and the Russian republic of Kalmykia (*2*) are well-known examples. Refugee children are a special risk category for infection with BBVs (*3*). When iatrogenic transmission was encountered in a pediatric infectious diseases clinic in Adelaide, South Australia, Australia, concern was raised about whether it was an isolated or a more widespread phenomenon.

The United Nations High Commissioner for Refugees estimates that there were 43.7 million forcibly displaced persons worldwide at the end of 2010, the highest number in 15 years. Of these, 27.5 million were internally displaced persons, 15.4 million were refugees, and 837,500 were asylum seekers (*4*). Children constituted more than half of the humanitarian refugee population in Australia (*5*). A refugee is legally defined as a person who is outside his or her country of nationality and is unable to return due to a well-founded fear of persecution because of race, religion, nationality, political opinion, or membership in a particular social group. By receiving refugee status, persons are guaranteed protection of their basic human rights and cannot be forced to return to a country where they fear persecution (*4*).

Australia receives refugees from all countries experiencing internal conflict. Some arriving refugees have parasite infestations and bacterial and viral infections, especially undiagnosed BBVs (*6*,^7^). During 2010–2011, a total of 13,799 persons were admitted under Australia’s Humanitarian Program.

The extent of the unusual problem of iatrogenic transmission of BBVs remains unknown because modes of transmission of individual cases are difficult to document. This report summarizes cases in 4 children from South Asia that illustrate the conditions extant in 1 city in Uzbekistan (Andijan), where medical procedures have resulted in transmission of HIV, hepatitis B virus (HBV), and hepatitis C virus (HCV). Among the case-patients are 2 children with BBV co-infection.

## Methods and Definitions

Detecting possible iatrogenic BBV infections in children relies on a careful history. However, accurate histories are difficult to obtain because, in many cases, details are acquired through interpreters and facts are lost in translation. Nevertheless, several encounters with a family, during which family members are encouraged to tell their life stories (*8*), usually results in an accurate medical history. For orphans, learning the mode of BBV acquisition usually is impossible, except in cases of maternal HIV-associated deaths and mother-to-child transmission. The Australian Paediatric Surveillance Unit collects data for all HIV-positive children in Australia and reports these data to the National HIV Registry. For HIV-positive children from high-risk countries whose mothers are known to be HIV negative, the information recorded does not indicate mode of transmission (compare surrogate breast-feeding); nevertheless, such cases should be considered suspicious in regard to the manner by which the virus was acquired. The Australian Paediatric Surveillance Unit has recorded a few cases of HIV in children from high-risk countries whose mothers were HIV negative, thus indicating the problem. Since data collection began in May 1993, a total of 77 HIV infections have been reported in children (*9*); 8 cases in children (<12 years of age) from high-prevalence countries were notified to the Australian National HIV Registry through the end of 2011. The mother of 1 child was reported as HIV negative. No information was available about the HIV status for the mothers of the 7 other children (A. McDonald, pers. comm.).

### Case Discovery

Refugee families undergo voluntary health screening after arrival. In South Australia, screening usually is conducted by the Migrant Health Unit of the South Australian Department of Health but sometimes by another health unit. Children in whom BBV infection is diagnosed are referred to the Women’s and Children’s Hospital (WCH) Infectious Diseases Clinic for assessment and management. Some children are not screened on arrival, and undiagnosed BBV infections may be discovered later.

### Iatrogenic BBV Infection

BBV infection acquired through injection of blood or blood products and for which vertical (mother-to-child) infection (including breast milk and surrogate breast-feeding) was ruled out by serologic testing of the mother and by history of sexual abuse and evidence of tattooing, piercing, and scarification. Iatrogenic infection, a form of horizontal transmission, usually involves mediation through injection of the BBV by a third party who might or might not be infected with the virus(es) in question. For this study, I defined an iatrogenic BBV infection according to all of the following criteria: BBV infection in a refugee child <18 years of age who was seen at the WCH Infectious Diseases Clinic during 2008–2010, who had been exposed to medical hollow-bore needles and blood products, who had acquired a BBV infection that was absent in his/her mother, and who had not been breast-fed by a surrogate or sexually abused but for whom other modes of horizontal infection could not be completely ruled out.

### Search Methods and Patients

Internet searches of databases, including PubMed and Google Scholar, were performed to obtain peer-reviewed and other journal articles by using the following search terms: iatrogenic blood-borne virus infection, refugee children, blood-borne viral infections in war and transition zones, HIV, HBV, HCV. In addition, case reports of 4 refugee children seen in the WCH pediatric infectious diseases clinic during January 2008–December 2010 (Table) are summarized to illustrate the insidious nature of BBV infection. These case-patients, included here with parental written consent, were victims of their country’s chaotic or absent infection control practices consequent to war and political strife.

**Table:** Presumptive iatrogenic BBV infections in refugee children from Uzbekistan, Australia, 2008–2010*

Case-patient	BBV Infection	Age, y/sex	Risk factor	Year of diagnosis	Maternal serostatus	Sibling serostatus
1	HIV/HBV	8/F	Blood transfusion, plasma transfusion	2010	Anti-HIV–, HBsAg+	Seronegative (2 sibs)
2†	HIV	9/M	Possible exposure to nonsterile injections	2010	Anti-HIV–	Anti-HBs+, anti-HCV+
3†	HCV	5/M	Possible exposure tononsterile injections	2010	Anti-HCV–	Anti-HIV+, anti-HCV–
4	HCV/HIV	6/M	Blood transfusion; IV; possible exposure to nonsterile injections	2008; 2012, respectively	Anti-HCV–, anti-HIV–	Anti-HCV–, anti-HIV– (1 sib)

*BBV, blood-borne virus; HBV, hepatitis B virus; HBsAg, hepatitis B surface antigen; +, positive; –, negative; HCV, hepatitis C virus; IV, intravenous. †Case-patients 2 and 3 are siblings.

## Case Reports

### Case-patient 1

An 8-year-old girl was brought for treatment in early 2010. She was born vaginally in a hospital in Uzbekistan after a full-term normal pregnancy. There were no neonatal concerns. At 18 months of age, severe hematemesis developed. Blood loss required admission to a clinic in Andijan and transfusions of donated plasma and of blood donated by her father. At 7 years of age, she underwent a tonsillectomy in the same clinic. She had no illness typical of HIV seroconversion. Her mother was hepatitis B surface antigen (HBsAg) positive and HIV negative. The girl’s 2 younger siblings were BBV negative but anti-HBs positive. As part of migrant health screening on arrival in Australia in 2009, family members underwent serologic tests for HIV, HBV, and HCV. The girl was anti-HIV-1 and HBsAg positive. Her HIV load was 1,010 HIV RNA copies/mL, and HBV DNA was not detectable (<20 IU/mL) (HBeAg and anti-HBe negative) in 2009. Her father was negative for BBV markers. Her CD4 count was 260 cells/μL (19%), indicating severe immunosuppression. Examination was largely unremarkable except for severe dental caries and an ulcer on the lower gums (PCR positive for herpes simplex type 1). She began antiretroviral therapy soon after diagnosis and has responded well.

### Case-patient 2

On arrival to Australia in 2010, a 9-year-old boy was discovered to be anti-HIV-1 positive on arrival. Both parents tested negative for anti-HIV-1 and anti-HCV. Through an interpreter, it was established that he had exposure to medical needles in the hospital. In 2004, he had undergone an appendectomy at the same Andijan clinic mentioned above; in summer 2008, he was admitted with “hepatitis” (presumably hepatitis A; see case-patient 3 below) to an Andijan hospital, where he received several injections (possibly vitamin K) by medical and nursing staff, but as far as is known, received no blood products and had no seroconverting-like illness. He had been asymptomatic. His prenatal history was unremarkable; he was born at term by normal vaginal delivery. He was vaccinated in Uzbekistan but to an unknown extent. He looked reasonably well. There was no lymphadenopathy. He had severe dental caries. He began antiretroviral therapy soon after his HIV-1 infection was diagnosed and has responded well.

### Case-patient 3

This child, the younger sibling of case-patient 2, arrived in Australia with his family in 2010 when he was 5 years 9 months of age. At arrival screening, he was anti-HIV-1 negative and anti-HCV positive. He had attended the clinic in Andijan in November 2007 for “management” of jaundice, presumably associated with hepatitis A infection acquired from his sibling (case-patient 2), who had contracted hepatitis A and received the described treatment ≈40 days before case-patient 3 received his. Case-patient 3 was in the Andijan clinic for 10 days and received daily injections (the nature of which is unknown)—presumably the source of the HCV infection. When first seen at WCH on referral, he was asymptomatic. There were no abnormal physical findings. He was repeatedly anti-HCV positive and HCV RNA negative, indicating resolved infection.

### Case-patient 4

This boy was born in Andijan in 2002. Immigration serology screening was not performed. He was first seen at WCH as a refugee in 2008 at 6 years of age after his general practitioner found him to be anti-HCV positive (anti-HIV-1 and HBsAg testing was not performed at the time). His mother was anti-HCV negative. He was born at 38 weeks’ gestation and had some neonatal problems for which he received multiple intravenous injections and blood transfusion(s) in an Andijan hospital. His 2 younger siblings were anti-HCV negative. Examination findings were normal except for serious dental caries requiring extractions. His liver function showed slightly elevated alanine aminotransferase (50 U/L [reference value 5–45 U/L]). Initially, nonquantitative HCV RNA was detectable in his peripheral blood, and in early 2011, his viral load was 623 IU/mL, at which time his liver function was entirely normal. In 2012, because of rising alanine aminotransferase (57 U/L) and increasing viral load (206,000 IU/mL), he was referred to a pediatric gastroenterologist, who noticed that anti-HIV and HBsAg/antibody tests had never been performed; results were positive for anti-HIV-1 (HBsAg and anti-HBs negative). The patient’s viral load was 18,300 RNA copies/mL. His CD4 count was 810 cells/μL (27%). Examination indicated no abnormality. His siblings and parents are anti-HIV negative.

## Discussion of Cases

Vertical transmission of the viruses involved in these 4 cases was ruled out by the mothers’ negative serostatus, as well as by exclusion based on history of surrogate breast-feeding and sexual abuse. Notwithstanding a small risk for horizontal transmission, the most likely mode of transmission is iatrogenic. Incomplete documentation made other cases speculative. One case-patient had an additional risk factor of rape. Three others with HBV infection in whom the serostatus of the mothers was tested and had revealed anti-HBs positivity could imply vertical transmission with maternal infection resolution and natural immunity. The presumptive cases demonstrate intended or unintended effects of nonfunctional infection control in the chaos left by political collapse and/or civil war on health systems and the blood supply. Such recent events have affected Central Asian countries, including Kazakhstan, Kyrgyzstan, Tajikistan, Turkmenistan, and to a lesser extent, Uzbekistan (*10*–*14*). Patients with speculative cases were from several sub-Saharan African countries, including Ethiopia, Kenya, Somalia, Liberia, and Ghana.

The extent of the problem remains unknown. A prospective study of 1,026 child refugees conducted in a tertiary pediatric health unit in Western Australia (March 2006– December 2008) showed an incidence of hepatitis B of 8.1% with no cases of HIV or HCV infection (*3*). The study did not report cases of iatrogenic transmission. The literature examining iatrogenic BBV infection is limited, but conflict and war clearly go hand in hand with increased prevalence of BBVs, especially in women and children. The cases reported here may represent the “tip of the iceberg.” Cases showing up in 1 city in Australia might or might not be an isolated event. The absence of conclusive data prevents a conclusion about whether these cases are isolated events or represent a widespread problem, but the cases provide a stimulus for reception countries to be alerted to a possible problem.

The 4 case-patients illustrate the price paid by children caught up in the effects of social disintegration. Such situations provide opportunities for sexually transmitted infections (including HIV) to increase in prevalence (through prostitution and intravenous/injection drug use) and therefore form an environment for possible iatrogenic transmission. Hamers and Downs (*15*) indicated the extent of the problem. Escalating intravenous/injection drug use, which highlights Central Asia’s geographic position along major drug-trafficking routes, has led to a corrupted blood supply. These upheavals seem to have formed the nidus of the documented epidemic of HIV in Central Asia (*16*). In Shymkent, 93 children contracted HIV either directly or indirectly through blood transfusion (*17*). Transmission of HIV to babies and children by medical personnel through unscreened blood transfusion has been reported in Kazakhstan and Kyrgyztan in 2007 and 2008, respectively (*18*).

Zahed (*3*) described the situation for children worldwide and pointed to Central Asia as a major problem area, particularly for spread of HIV infection. Thorne et al. (*16*) indicated that Uzbekistan is one of several hot spots for HIV transmission in Central Asia, with a few HIV cases in children having been reported, most of which resulted from nosocomial outbreaks in hospitals (*16*). No data indicate whether refugees are at higher or lower risk for BBV infection than others living in conflict zones. Nevertheless, the following modes of transmission could, arguably, inevitably increase the background prevalence of BBVs and, by inference, increase the risk for children living in these high-risk areas. Some of these children will reach reception countries as refugees or orphans.

## Modes of Transmission Contributing to Increased BBV Prevalence

### Contaminated Injections in Health Care Settings

According to the US Agency for International Development, “consecutive wars have made it nearly impossible to conduct effective and sustainable HIV prevention activities” (*19*). The World Health Organization estimated that 17%–19% of injections performed in sub-Saharan Africa during 2000 were administered unsafely (*20*). Simonsen et al. reviewed injection practices in the developing world with disturbing findings (*21*).

#### HIV and AIDS in Africa

Iatrogenic spread of HIV through inadequate screening facilities and improper use of needles and syringes by impoverished and undertrained health care workers puts patients at risk (*22*). Twenty percent of blood transfusions in 3 hospitals in Ashanti, Ghana, were unscreened (*23*). According to Purdin et al. (*24*), conflict and HIV dramatically intersect in sub-Saharan Africa. The 22.5 million HIV-infected persons in sub-Saharan Africa contain ≈90% of HIV-positive children and 68% of HIV-positive adults infected worldwide. Twenty-one sub-Saharan African countries have ongoing political conflict. Within their borders, as recorded in 2007, are 77% (1.6 million) of the world’s AIDS-related deaths (*25*–*27*).

Jenkins and Robalino (*28*) alluded to the hidden epidemic in Middle Eastern and North African countries affected by war and internal strife where no epidemiologic monitoring occurs and where iatrogenic transmission thus remain undetected. The rise in HIV prevalence for each country affected by poverty or internal political strife and dysfunctional medical services directly correlates with these situations (*28*).

#### HCV in Africa

In southern Cameroon, where west-central African chimpanzee strains of simian immunodeficiency virus, the source of HIV-1 group M, is prevalent among wild chimpanzees, ≈50% of some human birth cohorts were infected with HCV through unclear mechanisms (*29*) but suggesting high levels of iatrogenic transmission (*30*). Some have speculated that this level of iatrogenic transmission jump-started the HIV/AIDS pandemic through injection treatment of trypanosomiasis before 1951 (*31*). Excess deaths among trypanosomiasis patients treated before 1951 support this hypothesis (*32*).

### Unsafe Injections and Blood Transfusions

The pandemic of non-A, non-B hepatitis (now attributed to HCV infection) emerged in the second half of the 20th century. Almost certainly it was triggered and fed iatrogenically by the increasing use of injections and blood transfusion. In industrialized countries, the introduction of anti-HCV screening for blood donors sharply decreased the incidence of iatrogenic hepatitis C, but HCV continues to spread in developing countries, where the virus is still transmitted through unscreened blood transfusions and nonsterile injections (*33*). Even in countries without conflict, failure to protect the blood supply still can occur, In western India, 23 children with thalassemia were reported as being positive for HIV infection after receiving blood transfusions during January–August 2011 (*34*).

### Surrogate Breast-feeding and Feeding with Expressed Milk

In 2004, Shisana et al. (*35*) assessed blood and breast-milk exposures in children recruited from primary health clinics in the Free State province of South Africa and tested the children and their biological mothers for HIV infection. HIV positivity in children of HIV-negative mothers was associated with dental injections, surrogate breast-feeding, and feeding with expressed milk from a hospital milk room.

### Other Possibilities

No data exist on sharing of contaminated equipment during self-injection in children and adolescents in countries of high BBV prevalence (*36*). Also, tattoos, skin piercing, and scarification are common in Africa and are detectable on examination.

### Horizontal Infection Not Otherwise Specified

Information about modes of transmission in HIV-infected African children who have HIV-uninfected mothers is generally lacking. Since 1984, horizontally acquired HIV infection in African children who have HIV-negative mothers has been reported from 13 sub-Saharan countries (*37*). Injections related to health care provision or dental surgery (especially by informal providers) were more common in infected children, suggesting that horizontal HIV transmission through blood exposures is common in some sub-Saharan African countries.

In a 2005 serosurvey, Rehle et al. (*38*) estimated that the annual incidence of HIV infection was 0.5% in South African children 2–14 years of age. HIV infection in children who have HIV-negative mothers provided an estimate of horizontal HIV transmission. Medical injections, blood transfusion, and hospitalization almost certainly play a role; however, little is known about the extent and modes of horizontal HIV transmission in African children. Other contributors to the spread of BBVs include prostitution and rape in war; BBV-positive persons provide a link in the chain of infection that may lead to iatrogenesis.

## Implications and the Future

Estimates of up to 160,000 HIV, 4.7 million HCV, and 16 million HBV infections each year are attributable to unsafe injections (*39*). Developing world conflict and maldistribution of resources remain major contributors to the prevalence of BBV infection and affect the poor, the young, and the victims of rape in war. Preventing this is a challenge for resource-rich countries. A 2002 report that seems to have preceded the HIV epidemic in Central Asia, including Uzbekistan, indicated that the situation was ripe for an epidemic among children (*40*). This report illustrates the failure of United Nations to effectively address the drug problem and epidemics of BBVs. The “War on Drugs” has clearly failed and seems to enhance the effectiveness of drug barons and warlords in promulgating their trade. The practice of evidence-based medicine would resolve to abandon this wasteful and ill-conceived war. How industrialized countries can help is problematic. The World Health Organization has insufficient resources to monitor and maintain prevention programs. The affected countries have multitudinous difficulties, including providing food, water, and sanitation. Infection control competes with these and other priorities. The protection of vulnerable children thus remains unaddressed.
